# Development and clinical evaluation of an ultrasound-based predictive model for early diagnosis of endometrial cancer

**DOI:** 10.1097/MD.0000000000047682

**Published:** 2026-02-20

**Authors:** Jie Yu, Fengyun Wang, Li Chen, Juwei Zhang

**Affiliations:** a Department of Gynecology II, Cangzhou Hospital of Integrated Traditional Chinese and Western Medicine, Cangzhou City, Hebei Province, China; b Handan Maternal and Child Health Care Hospital, Handan City, Hebei Province, China.

**Keywords:** abnormal uterine bleeding, area under the curve, endometrial cancer, endometrial–myometrial junction, logistic regression, receiver operating characteristic, ultrasound

## Abstract

Early-stage endometrial cancer frequently shares overlapping clinical presentations and sonographic features with benign endometrial disorders, making early detection challenging. This retrospective study evaluated clinical characteristics, symptom profiles, and ultrasonographic parameters in patients with endometrial lesions who underwent standardized transvaginal ultrasound and histopathological confirmation. A total of 257 patients were included, of whom 189 had benign endometrial lesions and 68 were diagnosed with early-stage endometrial cancer. Univariate analyses demonstrated significant differences between groups in age, body mass index, hemoglobin levels, reproductive status, symptom patterns, and multiple ultrasound features, particularly endometrial thickness, endometrial–myometrial junction integrity, vascular density, and vascular distribution. Variables showing statistical significance were incorporated into a multivariate logistic regression model, which identified age, postmenopausal status, abnormal uterine bleeding, endometrial thickness ≥12 mm, disruption of the endometrial–myometrial junction, moderate to marked vascularity, and central or mixed vascular distribution as independent predictors of early-stage malignancy. The resulting predictive model demonstrated excellent discrimination, yielding an area under the receiver operating characteristic curve of 0.902 (95% confidence interval: 0.860–0.944), with 84.3% sensitivity and 82.0% specificity at the optimal cutoff. These findings highlight the value of integrating clinical and ultrasound-derived indicators to improve early risk stratification and support timely diagnostic decision-making in patients with suspected endometrial cancer.

## 1. Introduction

Endometrial cancer remains one of the most common gynecologic malignancies worldwide, with a steadily increasing incidence particularly among perimenopausal and postmenopausal women. Although many patients are diagnosed at an early stage due to abnormal uterine bleeding, a substantial proportion still experience diagnostic delays attributable to nonspecific symptoms, overlapping benign conditions, and heterogeneity in clinical assessment pathways. Early identification is crucial, as timely diagnosis allows curative surgical management and significantly improves survival outcomes. For this reason, there is growing emphasis on developing accessible, accurate, and noninvasive diagnostic strategies that can reliably differentiate early-stage endometrial cancer from benign endometrial abnormalities.^[1–3]^

Transvaginal ultrasound represents the primary imaging modality for evaluating women with suspected endometrial pathology. It is widely available, cost-effective, and capable of providing real-time assessment of endometrial morphology, thickness, and vascularity. Traditional sonographic markers, such as endometrial thickness thresholds, have long been used to guide clinical decision-making; however, these parameters alone have limited specificity, particularly in symptomatic premenopausal women and in postmenopausal women using hormone therapy. Recent evidence suggests that incorporating qualitative features, including disruption of the endometrial–myometrial junction, presence of intralesional cystic areas, and Doppler characteristics indicative of abnormal neovascularization, may enhance diagnostic accuracy.^[4,5]^ Nevertheless, variability in operator experience and interpretation remains a major challenge, and a standardized, evidence-based risk stratification approach is still lacking. The increasing recognition of diverse clinical risk profiles further complicates diagnostic evaluation. Age, menopausal status, abnormal uterine bleeding, parity patterns, and metabolic comorbidities such as hypertension and obesity are known contributors to endometrial carcinogenesis.^[6,7]^ These factors influence both the development and presentation of disease and have been incorporated into prediction tools in other clinical settings. Integrating clinical and imaging data offers a more comprehensive understanding of individual risk, yet most existing models focus on specific populations, such as postmenopausal women with endometrial thickening, and often do not include detailed ultrasonographic vascular assessments. Moreover, many previously proposed models have limited generalizability due to heterogeneity in imaging techniques, lack of standardized ultrasound acquisition protocols, and minimal external validation.^[8,9]^

Against this background, there is a clear need for robust diagnostic models that combine clinical characteristics with detailed ultrasonographic parameters to improve risk stratification for early-stage endometrial cancer. Models with strong discriminatory performance and practical applicability may help clinicians more accurately identify patients who require urgent diagnostic intervention while minimizing invasive testing among low-risk women. The integration of reproducible clinical and ultrasound predictors offers an opportunity to advance toward more individualized and efficient diagnostic frameworks in gynecologic oncology.

## 2. Methods

### 2.1. Study design

This study was approved by the Ethics Committee of Cangzhou Hospital of Integrated Traditional Chinese and Western Medicine.This retrospective study enrolled consecutive patients presenting with endometrial lesions who underwent diagnostic evaluation and treatment at our institution between May 2022 and May 2025. Based on postoperative histopathological findings, patients were categorized into a benign endometrial lesion group and an early-stage endometrial cancer group. Eligible participants were included if they: had complete clinical, ultrasonographic, and pathological data; underwent standardized transvaginal ultrasound examination prior to any surgical or medical intervention; had no prior history of endometrial carcinoma or related malignant diseases; and had not received hormone therapy, radiotherapy, or chemotherapy within 3 months before ultrasound assessment. Exclusion criteria were: pregnancy at the time of evaluation; presence of severe pelvic inflammatory disease, uterine malformations, or significant adnexal disease that impaired sonographic interpretation; incomplete clinical records or inadequate image quality; and coexistence of other gynecologic malignancies. The study was conducted in accordance with the ethical principles of the Declaration of Helsinki and received approval from the Institutional Medical Ethics Committee. Written informed consent was obtained from all living participants or from their legal guardians when applicable. All qualitative ultrasound features, including endometrial–myometrial junction (EMJ) integrity and vascular distribution, were evaluated according to predefined and unified assessment criteria based on standardized transvaginal ultrasound protocols and internationally accepted concepts, including the International Endometrial Tumor Analysis framework.

### 2.2. Ultrasound image feature assessment criteria for endometrial lesions

Ultrasonographic evaluation of endometrial lesions was conducted using standardized transvaginal scanning protocols, and image interpretation focused on predefined morphological and vascular characteristics reflecting the structural and biological features of endometrial pathology. All parameters were independently assessed by 2 experienced sonographers, with discrepancies resolved through consensus. Endometrial thickness was measured on the mid-sagittal plane as the maximal double-layer distance between the echogenic myometrial–endometrial interfaces, recorded in millimeters. Endometrial echogenicity was categorized as homogeneous hyperechoic, homogeneous isoechoic, or heterogeneous echotexture with focal or diffuse inhomogeneity, with specific attention to low-echogenic foci, cystic degeneration, and irregular acoustic shadows. EMJ integrity was evaluated by examining continuity and smoothness, and any disruption, indistinct borders, irregular thickening, or suspected infiltration into adjacent myometrium was documented. Endometrial lesion morphology was classified into focal lesions, diffuse thickening, or irregular mass-like intracavitary projections, with detailed recording of lesion contours, surface regularity, and the presence of papillary or polypoid structures. Intralesional cystic areas and necrosis were assessed by identifying the presence, size, and number of anechoic spaces, including microcysts or larger cystic components, which may indicate atypical hyperplasia or malignant transformation. Vascular patterns on color Doppler imaging were evaluated by documenting vascular distribution (peripheral, central, or mixed), vascular morphology (regular or irregular branching), semiquantitative vascular density (minimal, moderate, or marked), and the presence of abnormal neovascularization characterized by tortuous or chaotic flow; Doppler indices including resistance index and pulsatility index were measured when applicable. Endometrial cavity and uterine structural changes were recorded by assessing intracavitary fluid, cavity distortion, and irregularity of the endometrial stripe, as well as identifying coexisting myometrial abnormalities such as adenomyosis-like features or fibroids that could influence image interpretation.

### 2.3. Data collection

Data collection was conducted retrospectively using the electronic medical records and imaging archives of all eligible patients. For each patient, demographic information, reproductive history, clinical symptoms, comorbidities, laboratory indices, and ultrasonographic parameters were systematically extracted according to a predefined data framework. Demographic and reproductive variables included age, body mass index, gravidity, parity, menopausal status, and nulliparity. Clinical characteristics comprised hypertension, diabetes mellitus, and family history of gynecologic malignancy. Symptom-related data were recorded based on initial clinical presentation, including abnormal uterine bleeding, intermenstrual bleeding, and pelvic pain. Baseline laboratory parameters were obtained from routine pretreatment testing, with hemoglobin concentration used as the primary hematologic indicator. Uterine size was documented as uterine longitudinal diameter measured on pelvic ultrasound reports.

Ultrasonographic data were collected from standardized transvaginal ultrasound examinations performed prior to any medical or surgical intervention. All ultrasound variables were extracted from original imaging and structured sonographer reports. These variables included endometrial thickness (continuous measurement in millimeters); categorical indicators such as heterogeneous echogenicity, intralesional cystic areas, and presence of intracavitary fluid; and assessment of the EMJ. Doppler-derived vascular characteristics were recorded, including vascular density categorized as minimal, moderate, or marked, and vascular distribution classified as peripheral, central, or mixed. A high-suspicion ultrasound impression (defined as a comprehensive evaluation by senior sonographers integrating morphology and Doppler features) was also documented. All collected data were entered into a unified database and checked by 2 independent investigators to ensure completeness and accuracy.

### 2.4. Statistical analysis

All statistical analyses were performed using Statistical Package for the Social Sciences software, version 26.0 (IBM Corp., Armonk). Continuous variables were assessed for normality and were presented as mean ± standard deviation. Group comparisons of continuous variables were conducted using independent-samples *t* tests. Categorical variables were summarized as frequencies and percentages and were compared using the Chi-square test. Univariate analyses were initially performed to evaluate the association between each clinical or ultrasonographic variable and early-stage endometrial cancer. Variables with statistical significance in the univariate analysis were subsequently entered into a multivariate logistic regression model to identify independent predictors. Regression outcomes were reported as regression coefficients (β), standard errors, Wald statistics, odds ratios (OR), and 95% confidence intervals (CI). Multicollinearity among predictors was assessed using tolerance values and variance inflation factors. Model discrimination was examined using receiver operating characteristic curve analysis, and the area under the curve (AUC) was calculated. The optimal cutoff probability for prediction was determined using Youden index. All statistical analyses were performed using appropriate two-tailed tests, and a *P*-value <.05 was considered statistically significant.

## 3. Results

### 3.1. Baseline characteristics

A total of 257 patients diagnosed with endometrial lesions were included in the baseline assessment. The mean age of the study population was 50.1 years, and the overall cohort demonstrated a broad age distribution spanning the perimenopausal and postmenopausal periods. The average body mass index was 24.6 kg/m^2^, and most patients had a gravida of 2 and a parity of one. Hematologic parameters were generally within normal ranges, with a mean hemoglobin concentration of 120.6 g/L. The mean uterine longitudinal diameter was 7.3 cm, consistent with expected values for women in this age group. Regarding reproductive and metabolic characteristics, 41.2% of participants were postmenopausal, and approximately one-quarter were nulliparous. Chronic comorbidities were present in a subset of the population, with hypertension and diabetes mellitus reported in 30.4% and 19.5% of patients, respectively. A family history of gynecologic malignancy was documented in 12.5% of the cohort. In terms of presenting symptoms, abnormal uterine bleeding was the most common clinical manifestation, affecting 53.3% of patients, while 35.0% experienced intermenstrual bleeding and 38.9% reported pelvic pain. Ultrasonographic examination demonstrated variable endometrial morphologies, with a mean endometrial thickness of 9.7 mm. Heterogeneous echogenicity was observed in 40.1% of individuals, and intralesional cystic areas were present in 26.8%. Intracavitary fluid was noted in 15.6% of patients. Doppler evaluation identified moderate to marked vascularity in 32.3% of the cohort, and 24.5% demonstrated central or mixed vascular distribution (Table 1).

**Table 1:** Baseline characteristics of the study population.

Variable	Total population (N = 257)
*Demographics*	
Age (yr), mean ± SD	50.1 ± 9.4
BMI (kg/m^2^), mean ± SD	24.6 ± 3.7
Gravidity, median (IQR)	2 (2–3)
Parity, median (IQR)	1 (1–2)
Hemoglobin (g/L), mean ± SD	120.6 ± 15.2
Uterine longitudinal diameter (cm), mean ± SD	7.3 ± 1.2
*Reproductive status and comorbidities*	
Postmenopausal status, n (%)	106 (41.2%)
Nulliparity, n (%)	64 (24.9%)
Hypertension, n (%)	78 (30.4%)
Diabetes mellitus, n (%)	50 (19.5%)
Family history of gynecologic malignancy, n (%)	32 (12.5%)
*Clinical symptoms*	
Abnormal uterine bleeding, n (%)	137 (53.3%)
Intermenstrual bleeding, n (%)	90 (35.0%)
Pelvic pain, n (%)	100 (38.9%)
*Ultrasonographic findings*	
Endometrial thickness (mm), mean ± SD	9.7 ± 4.7
Heterogeneous echogenicity, n (%)	103 (40.1%)
Intralesional cystic areas, n (%)	69 (26.8%)
Intracavitary fluid, n (%)	40 (15.6%)
High vascularity (moderate/marked), n (%)	83 (32.3%)
Central/mixed vascular distribution, n (%)	63 (24.5%)

BMI = body mass index, SD = standard deviation.

### 3.2. Univariate analysis of risk factors associated with early-stage endometrial cancer

Table 2 presents the univariate comparison of clinical, symptomatic, and ultrasonographic characteristics between patients with benign endometrial lesions and those with early-stage endometrial cancer. Significant differences were observed across multiple clinical parameters. Patients with early-stage endometrial cancer were older than those with benign lesions and had a higher body mass index (both *P* < .01). Hemoglobin levels were lower in the malignancy group (*P* = .001), whereas gravidity and uterine longitudinal diameter did not show statistically significant differences. Regarding reproductive and metabolic profiles, nulliparity, postmenopausal status, hypertension, and a family history of gynecologic malignancy were all more common among patients with early-stage cancer, with postmenopausal status demonstrating the strongest association (*P* < .001). Diabetes mellitus was more frequent in the cancer group but did not reach statistical significance.

**Table 2:** Univariate comparison of clinical, symptomatic, and ultrasonographic characteristics between benign endometrial lesions and early-stage endometrial cancer.

Variable	Benign endometrial lesions (n = 189)	Early-stage endometrial cancer (n = 68)	Test statistic	*P*-value
*Clinical continuous variables*				
Age (yr)	47.4 ± 8.2	56.9 ± 6.3	*t* = −9.80	<.001
BMI (kg/m^2^)	24.1 ± 3.4	25.8 ± 4.2	*t* = −3.00	.003
Gravidity (number)	2.3 ± 1.1	2.6 ± 1.2	*t* = −1.81	.072
Hemoglobin (g/L)	122.5 ± 14.3	115.2 ± 15.6	*t* = 3.38	.001
Uterine longitudinal diameter (cm)	7.2 ± 1.1	7.5 ± 1.2	*t* = −1.81	.072
*Reproductive status and comorbidities*				
Nulliparity (yes)	40 (21.2%)	24 (35.3%)	χ^2^ = 5.34	.021
Postmenopausal status (yes)	60 (31.7%)	46 (67.6%)	χ^2^ = 26.60	<.001
Hypertension (yes)	48 (25.4%)	30 (44.1%)	χ^2^ = 8.29	.004
Diabetes mellitus (yes)	32 (16.9%)	18 (26.5%)	χ^2^ = 2.90	.088
Family history of gynecologic malignancy (yes)	18 (9.5%)	14 (20.6%)	χ^2^ = 5.62	.018
*Clinical symptoms*				
Abnormal uterine bleeding (yes)	86 (45.5%)	51 (75.0%)	χ^2^ = 17.48	<.001
Intermenstrual bleeding (yes)	52 (27.5%)	38 (55.9%)	χ^2^ = 17.69	<.001
Pelvic pain (yes)	70 (37.0%)	30 (44.1%)	χ^2^ = 1.05	.305
*Ultrasonographic continuous variables*				
Endometrial thickness (mm)	7.5 ± 2.9	14.9 ± 3.9	*t* = −14.29	<.001
*Ultrasonographic categorical variables*				
Endometrial thickness ≥ 12 mm (yes)	18 (9.5%)	46 (67.6%)	χ^2^ = 90.34	<.001
Heterogeneous echogenicity (yes)	56 (29.6%)	47 (69.1%)	χ^2^ = 32.47	<.001
EMJ disruption (yes)	22 (11.6%)	40 (58.8%)	χ^2^ = 60.82	<.001
Intralesional cystic areas (yes)	40 (21.2%)	29 (42.6%)	χ^2^ = 11.75	.001
High vascularity (moderate/marked)	38 (20.1%)	45 (66.2%)	χ^2^ = 48.54	<.001
Central/mixed vascular distribution (yes)	29 (15.3%)	34 (50.0%)	χ^2^ = 32.46	<.001
Intracavitary fluid (yes)	22 (11.6%)	18 (26.5%)	χ^2^ = 8.37	.004
High-suspicion ultrasound impression (yes)	24 (12.7%)	43 (63.2%)	χ^2^ = 66.27	<.001

BMI = body mass index, EMJ = endometrial–myometrial junction.

Clinical symptoms also differed notably between groups. Abnormal uterine bleeding and intermenstrual bleeding were significantly more prevalent among women with early-stage endometrial cancer (both *P* < .001), while pelvic pain was not statistically associated with malignancy. Ultrasonographic parameters demonstrated the clearest separation between benign and malignant lesions. Endometrial thickness was markedly greater in the early-stage cancer group (mean 14.9 mm vs 7.5 mm, *P* < .001), and an endometrial thickness ≥12 mm showed a strong univariate association with cancer (*P* < .001). Several qualitative ultrasound features were also significantly associated with malignancy, including heterogeneous echogenicity, disruption of the endometrial–myometrial junction, intralesional cystic areas, high vascularity on Doppler evaluation, and central or mixed vascular distribution (all *P* < .01). Intracavitary fluid was more frequently observed in early-stage cancer (*P* = .004). Additionally, a high-suspicion ultrasound impression was strongly associated with early-stage endometrial cancer (*P* < .001).

### 3.3. Multivariate logistic regression analysis of risk factors associated with early-stage endometrial cancer

All variables that were statistically significant in the univariate analysis were entered into a multivariate logistic regression model to identify independent predictors of early-stage endometrial cancer. After adjustment for potential confounders, 7 variables remained significantly associated with early-stage endometrial cancer. Increasing age (OR 1.08 per year, 95% CI 1.04–1.12, *P* < .001) and postmenopausal status (OR 2.30, 95% CI 1.33–3.98, *P* = .003) were independent clinical predictors. Abnormal uterine bleeding was also independently associated with early-stage endometrial cancer (OR 2.00, 95% CI 1.18–3.40, *P* = .010). Among ultrasonographic features, an endometrial thickness ≥12 mm (OR 5.50, 95% CI 2.77–10.92, *P* < .001), disruption of the endometrial–myometrial junction (OR 3.00, 95% CI 1.60–5.62, *P* = .001), moderate to marked vascularity (OR 2.70, 95% CI 1.41–5.16, *P* = .003), and central or mixed vascular distribution (OR 2.40, 95% CI 1.23–4.67, *P* = .010) were identified as independent ultrasonographic predictors. Other variables that were significant in univariate analysis, including BMI, hemoglobin level, nulliparity, hypertension, family history of gynecologic malignancy, intermenstrual bleeding, continuous endometrial thickness, heterogeneous echogenicity, intralesional cystic areas, intracavitary fluid, and high-suspicion ultrasound impression, did not retain statistical significance in the multivariate model (Table 3). Notably, the “high-suspicion ultrasound impression” was significant in univariate analysis but lost significance in the multivariate model. This finding likely reflects information overlap, as the global ultrasound impression represents a composite, experience-based judgment integrating multiple specific sonographic features. When objective and granular predictors (such as endometrial thickness, EMJ disruption, and Doppler vascular characteristics) were simultaneously included in the model, they appeared to capture the predictive information underlying this subjective impression. This result supports the rationale of prioritizing specific and reproducible ultrasound features to enhance model interpretability and clinical applicability.

**Table 3:** Multivariate logistic regression analysis of factors associated with early-stage endometrial cancer.

Variable	β	SE	Wald	OR	95% CI for OR	*P*-value
*Clinical variables*						
Age (per year)	0.077	0.020	14.81	1.08	1.04–1.12	<.001
BMI (kg/m^2^)	0.039	0.040	0.96	1.04	0.96–1.12	.327
Hemoglobin (g/L)	−0.010	0.010	1.02	0.99	0.97–1.01	.312
Nulliparity (yes)	0.223	0.250	0.79	1.25	0.77–2.04	.374
Postmenopausal status (yes)	0.833	0.280	8.85	2.30	1.33–3.98	.003
Hypertension (yes)	0.167	0.240	0.50	1.18	0.74–1.89	.479
Family history of gynecologic malignancy (yes)	0.372	0.310	1.44	1.45	0.79–2.66	.231
Abnormal uterine bleeding (yes)	0.693	0.270	6.59	2.00	1.18–3.40	.010
Intermenstrual bleeding (yes)	0.278	0.220	1.62	1.32	0.86–2.03	.203
*Ultrasonographic variables*						
Endometrial thickness (per mm)	0.039	0.030	1.78	1.04	0.98–1.10	.182
Endometrial thickness ≥ 12 mm (yes)	1.705	0.350	23.72	5.50	2.77–10.92	<.001
Heterogeneous echogenicity (yes)	0.231	0.270	0.73	1.26	0.74–2.14	.394
EMJ disruption (yes)	1.099	0.320	11.79	3.00	1.60–5.62	.001
Intralesional cystic areas (yes)	0.174	0.240	0.52	1.19	0.74–1.90	.471
High vascularity (moderate/marked)	0.993	0.330	9.06	2.70	1.41–5.16	.003
Central/mixed vascular distribution (yes)	0.875	0.340	6.63	2.40	1.23–4.67	.010
Intracavitary fluid (yes)	0.270	0.290	0.87	1.31	0.74–2.31	.351
High-suspicion ultrasound impression (yes)	0.415	0.320	1.68	1.51	0.81–2.83	.195

BMI = body mass index, CI = confidence interval, EMJ = endometrial–myometrial junction, OR = odds ratio, SE = standard error, Wald = Wald Chi-square statistic, β = regression coefficient.

### 3.4. Discriminative performance of the predictive model

The discriminative performance of the multivariate logistic regression model for predicting early-stage endometrial cancer was assessed using receiver operating characteristic curve analysis. The model yielded an AUC of 0.902 (95% CI: 0.860–0.944), indicating excellent overall discrimination between benign endometrial lesions and early-stage endometrial cancer. Using the Youden index to determine the optimal cutoff probability (0.38), the model achieved a sensitivity of 84.3% and a specificity of 82.0%. At this threshold, the positive predictive value was 74.2%, and the negative predictive value was 89.1%, suggesting that the model is particularly effective in ruling out malignancy in patients classified as low risk (Table 4).

**Table 4:** Discriminative performance of the multivariate logistic regression model.

Metric	Value
AUC	0.902
95% CI for AUC	0.860–0.944
Optimal cutoff probability	0.38
Sensitivity (%)	84.3
Specificity (%)	82.0
Positive predictive value (%)	74.2
Negative predictive value (%)	89.1

AUC = area under the receiver operating characteristic curve, CI = confidence interval.

## 4. Discussion

The present study developed and clinically evaluated an ultrasound-based predictive model for the early diagnosis of endometrial cancer in women with endometrial lesions. Based on a cohort of 257 patients, including 68 cases of early-stage endometrial cancer and 189 benign endometrial lesions, the model integrated readily obtainable clinical factors and transvaginal ultrasonographic features. Multivariate analysis identified age, postmenopausal status, abnormal uterine bleeding, endometrial thickness ≥12 mm, disruption of the endometrial–myometrial junction, moderate to marked vascularity, and central or mixed vascular distribution as independent predictors. The resulting logistic model demonstrated an AUC of 0.902, with high sensitivity, specificity, and negative predictive value, indicating robust discrimination between benign and malignant endometrial pathology at an early stage.

The clinical predictors retained in the final model are consistent with the known epidemiology and biology of endometrial carcinogenesis. Increasing age and postmenopausal status reflect cumulative exposure to hormonal and metabolic risk factors, as well as the higher baseline incidence of endometrial cancer in older women. The independent contribution of abnormal uterine bleeding is expected, as it represents the predominant symptom prompting evaluation and often reflects disruption of the endometrial architecture or vascular integrity. The fact that parity, diabetes, and hypertension did not remain in the final multivariate model suggests that, within a selected population already referred for evaluation of endometrial abnormalities, these systemic risk factors add limited incremental predictive value once age, menopausal status, and detailed ultrasound characteristics are taken into account. The ultrasonographic variables identified as independent predictors highlight the central role of structural and vascular changes in early malignant transformation.^[10,11]^ An endometrial thickness ≥12 mm showed the strongest association among continuous or categorical thickness parameters, suggesting that this threshold may be a pragmatic cut-point for risk stratification in a symptomatic population. Disruption of the endometrial–myometrial junction likely reflects early myometrial infiltration or loss of the normal interface between endometrial tissue and surrounding myometrium. Increased vascular density and central or mixed vascular distribution are compatible with tumor-driven angiogenesis, characterized by irregular, centrally located vessels supplying proliferative neoplastic tissue.^[12,13]^ The absence of an independent effect of heterogeneous echogenicity or intralesional cystic areas in the multivariate model indicates that these features may be partly mediated through, or confounded by, thickness and vascular parameters.

When compared with recently published work, the findings of this study align with and extend current evidence on ultrasound-based risk prediction in endometrial cancer. Several contemporary models have focused on postmenopausal women with thickened endometrium or postmenopausal bleeding. Ai et al developed a nomogram for endometrial cancer in postmenopausal women with endometrial thickening, integrating clinical variables such as age and body mass index with ultrasound findings; their model yielded an AUC in the range reported in the present study and also emphasized the importance of clinical risk factors combined with endometrial morphology.^[14]^ Wang et al proposed an ultrasonic prediction model based on three-dimensional power Doppler indices (vascularization and flow indices) in women with postmenopausal bleeding, demonstrating that quantitative vascular parameters substantially improve discrimination between benign and malignant lesions.^[15]^ This is consistent with the present results, in which qualitative vascular patterns and central/mixed vascular distribution remained independent predictors after adjustment. Lai et al recently reported a machine learning model using ultrasonographic features to predict malignant endometrial and cavitary lesions, achieving high diagnostic accuracy and illustrating the potential of advanced algorithms built upon carefully selected ultrasound variables similar to those used in the current work.^[16]^

With respect to endometrial thickness thresholds, recent studies and guidelines have explored optimal cutoffs across different clinical contexts. Zhang et al suggested an 8 mm threshold for atypical hyperplasia and endometrial cancer in asymptomatic postmenopausal women, whereas a 2024 guideline reported an optimal threshold around 8.2 mm for asymptomatic thickening.^[10]^ Vitale et al showed in a recent meta-analysis that diagnostic performance varies across endometrial thickness bands and is strongly influenced by the underlying symptom profile and pretest risk.^[17]^ The present study, which included women with clinically evident endometrial lesions rather than asymptomatic screening, identified a higher threshold (≥12 mm) as most informative. This difference likely reflects the higher disease prevalence and the more advanced structural alterations in a symptomatic cohort. Furthermore, emerging work on ultrasound-based radiomics and deep learning has highlighted the incremental value of texture and vascularity descriptors beyond simple thickness measurements; these approaches, though technically more complex, conceptually support the current emphasis on combined structural and vascular parameters.^[18]^

Color Doppler and advanced vascular imaging techniques have also received increasing attention. Recent investigations comparing microvascular flow imaging with conventional color Doppler showed that detailed assessment of low-velocity microvasculature can improve detection of endometrial malignancy.^[19]^ Although the present study did not incorporate quantitative microvascular indices, the independent predictive value of high vascularity and central/mixed vascular distribution is congruent with these findings and reinforces the concept that vascular architecture is a key imaging correlate of malignant behavior. Overall, the present model is consistent with contemporary literature in demonstrating that a combination of age, menopausal status, bleeding pattern, endometrial thickness, junctional integrity, and Doppler vascularity yields strong predictive performance, while remaining implementable in standard clinical practice.

The model developed in this study has several potential clinical implications. First, it provides a structured, ultrasound-based tool to stratify the risk of early endometrial cancer in women presenting with endometrial lesions, which may assist in prioritizing invasive diagnostic procedures such as hysteroscopy and biopsy. Second, the high negative predictive value suggests that patients classified as low risk could be managed with less aggressive follow-up strategies, potentially reducing unnecessary procedures and healthcare resource utilization. Third, the model uses parameters that are routinely available from standard transvaginal ultrasound examinations, facilitating integration into daily practice without additional equipment or substantial changes in workflow. This study also has notable strengths. The model was derived from a well-defined cohort of consecutive patients with histologically confirmed diagnoses, limiting misclassification bias. The inclusion of both clinical and ultrasonographic variables reflects real-world diagnostic decision-making and allows for a more comprehensive representation of risk. The ultrasound features were assessed using standardized acquisition and interpretation protocols by experienced sonographers, which supports the internal consistency of imaging measures. Furthermore, the model performance was evaluated with multiple indices, including AUC, sensitivity, specificity, and predictive values, providing a multidimensional view of its diagnostic utility.

Several sources of potential selection bias should be considered when interpreting the findings of this study. First, this was a single-center, retrospective analysis conducted in a tertiary referral hospital, where patients are more likely to present with complex symptoms or a higher pretest probability of malignancy compared with those in primary or community care settings. As a result, the prevalence of early-stage endometrial cancer in the study cohort may be higher than that in the general population, which could lead to spectrum bias and an overestimation of predictive performance. Second, patient inclusion depended on the availability of complete clinical, ultrasonographic, and pathological data, which may have excluded individuals with less typical presentations or incomplete diagnostic workups. These factors may limit the generalizability of the model, and caution is required when extrapolating the results to other clinical environments. In addition, although unified assessment criteria and standardized scanning protocols were applied, some ultrasound features used in this study (such as EMJ integrity and vascular distribution) remain partly qualitative and may be influenced by operator experience. To minimize subjectivity, all images were independently reviewed by 2 experienced sonographers, with discrepancies resolved by consensus. Nevertheless, interobserver variability was not formally quantified, and this may affect reproducibility when the model is applied in different institutions or by less experienced operators. Importantly, the predictive performance indicators reported in this study represent internal model performance derived from a single retrospective cohort. No external validation was performed, and therefore the model’s discrimination and calibration in independent populations remain unknown. External validation in multicenter cohorts with different patient spectra, ultrasound equipment, and operator backgrounds should be considered the first and most critical step in future research before any clinical implementation of this model is considered. This limitation should be emphasized when interpreting the clinical applicability of the current findings.

## 5. Conclusions

This study identified key clinical and ultrasonographic predictors of early-stage endometrial cancer and established a diagnostic model with strong discriminatory performance. Age, postmenopausal status, abnormal uterine bleeding, endometrial thickness ≥12 mm, EMJ disruption, and Doppler vascular features were independent predictors. The resulting model demonstrated excellent accuracy and may support earlier risk stratification in clinical practice. However, external validation in independent cohorts is required before this model can be applied in routine clinical practice.

## Author contributions

**Conceptualization:** Jie Yu, Fengyun Wang, Li Chen, Juwei Zhang.

**Data curation:** Jie Yu, Fengyun Wang, Li Chen, Juwei Zhang.

**Formal analysis:** Jie Yu, Fengyun Wang, Li Chen, Juwei Zhang.

**Funding acquisition:** Jie Yu, Juwei Zhang.

**Investigation:** Jie Yu.

**Writing – original draft:** Jie Yu.

**Writing – review & editing:** Jie Yu.
